# Association between weight-adjusted waist index and C-reactive protein in adults: A population-based study

**DOI:** 10.1097/MD.0000000000049044

**Published:** 2026-05-22

**Authors:** Qiaoli Liu, Caifeng Gu, Xiaofeng Zhang, Haifeng Miao, Changcai Wu, Piao Hu, Fei Wu

**Affiliations:** a Department of Infectious Diseases, Xiaoshan Affiliated Hospital of Wenzhou Medical University, Hangzhou, Zhejiang, China; b Department of Cardiovascular Medicine, Heze Luxin Hospital, Heze, Shandong, China; c Department of General Practice, Kanshan Branch of the First People’s Hospital of Xiaoshan District Medical Consortium, Hangzhou, Zhejiang, China; d Department of General Practice, Xiaoshan Affiliated Hospital of Wenzhou Medical University, Hangzhou, Zhejiang, China.

**Keywords:** CRP, NHANES, WWI

## Abstract

The weight-adjusted waist index (WWI) and C-reactive protein (CRP) have been independently associated with multiple chronic conditions. Given that central adiposity drives systemic inflammation, and WWI serves as a novel marker of central obesity, WWI may be associated with CRP levels. However, evidence on this relationship remains limited. This study aimed to explore the association between WWI and CRP in a nationally representative sample of US adults. This cross-sectional study included 8495 participants from the National Health and Nutrition Examination Survey. Weighted multiple regression and subgroup analyses were conducted to assess the association between WWI and CRP, adjusting for potential confounders. Generalized additive models and 2-piecewise linear regression were used to explore nonlinear relationships. After adjusting for potential confounders, a positive association was observed between WWI and CRP (β = 1.060, 95% confidence interval [CI] = 0.652–1.468). Subgroup analyses further revealed a significant positive association, particularly among participants with coronary heart disease (β = 4.357, 95% CI = 1.461–7.253), diabetes mellitus (β = 2.282, 95% CI = 1.031–3.532), or heavy drinking (β = 1.728, 95% CI = 0.758–2.698), as well as in non-Hispanic Black individuals (β = 1.650, 95% CI = 0.949–2.351). Nonlinear relationships were observed in current smokers and adults aged 20 to 60 years, with inflection points at 12.349 cm/√kg and 11.514 cm/√kg, respectively. The results indicate that higher WWI above the identified thresholds was associated with elevated CRP levels, particularly among current smokers and adults aged 20 to 60 years. These findings suggest that WWI may serve as a simple anthropometric indicator for identifying individuals with increased inflammatory risk. Further prospective studies are needed to validate these findings.

## 1. Introduction

C-reactive protein (CRP) is an acute-phase protein and plays a pivotal role in innate immunity. Its serum concentration increases significantly within 6 to 8 hours following inflammatory stimulation, reaching peak levels within 24 to 48 hours, and demonstrates a half-life of approximately 19 hours.^[1]^ Despite traditionally being classified as an acute-phase reactant, CRP levels can also be elevated in various chronic conditions, including cardiovascular diseases, diabetes mellitus, stroke, hypertension, nonalcoholic fatty liver disease, and related metabolic diseases.^[1–4]^ Moreover, CRP has emerged as a significant biomarker associated with these chronic conditions,^[1–4]^ highlighting its broader role in health beyond acute inflammation.^[5]^ Accumulating evidence has established inflammation as a critical pathogenic mechanism driving the progression of numerous chronic diseases.^[6]^

Traditionally, body mass index (BMI) and waist circumference (WC) have been used to assess obesity.^[7]^ However, both measures fail to distinguish between lean mass and fat mass, raising concerns about their accuracy in recent years.^[8,9]^ In response, Park et al proposed the weight-adjusted waist index (WWI) in 2018, which is calculated by dividing WC in centimeters by the square root of body weight in kilograms.^[10]^ This new index seeks to maximize the benefits of WC and lessen its association with BMI, delivering a more accurate evaluation of visceral fat and metabolic risk.^[11,12]^ Emerging evidence suggests that WWI, as a novel obesity index, may offer superior insights into visceral fat accumulation and its metabolic consequences compared with traditional measures like BMI and WC.^[12,13]^

Obesity, characterized by excessive fat accumulation in adipose tissue, is a significant risk factor for the development of chronic medical conditions, including type 2 diabetes mellitus, hypertension, atherosclerotic cardiovascular diseases, nonalcoholic fatty liver disease, and a spectrum of associated comorbidities.^[14]^ Notably, WWI has been independently associated with the development and progression of these obesity-related chronic conditions.^[12,13,15]^ Currently, obesity is a well-sestablished contributor to systemic inflammation and elevated CRP levels.^[16,17]^ CRP is primarily synthesized by hepatocytes upon stimulation by pro-inflammatory cytokines and is also expressed by adipocytes, indicating a direct link between chronic inflammation and obesity.^[18,19]^ This relationship highlights the importance of investigating obesity-related indices, such as the WWI, in understanding chronic inflammation. Given the established role of visceral fat in promoting systemic inflammation, WWI could serve as a valuable tool for understanding the relationship between obesity and chronic inflammation.

Although accumulating evidence has demonstrated associations between WWI and CRP with various chronic health outcomes, the underlying relationship between these 2 biomarkers remains poorly understood. This study aimed to elucidate the association between WWI and CRP. The findings may provide a basis for further research into the mechanisms of chronic inflammation and could help generate hypotheses for future clinical intervention strategies.

## 2. Materials and methods

### 2.1. Study population

**Figure 1.**
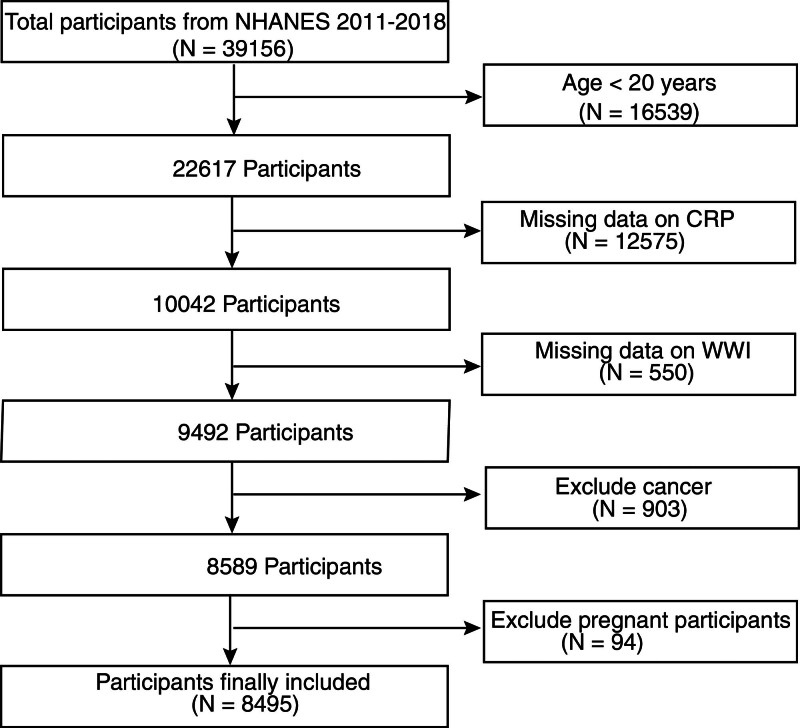
Flowchart of participant selection. CRP = C-reactive protein, NHANES = National Health and Nutrition Examination Survey, WWI = weight-adjusted waist index.

Stratified multistage probability sampling was employed in the National Health and Nutrition Examination Survey to create a representative sample for accurately assessing the health and nutritional status of the US population. Data were collected from 4 cycles for analysis in this study: 2011 to 2012, 2013 to 2014, 2015 to 2016, and 2017 to 2018. Among the 39,156 cases, we excluded those younger than 20 years (n = 16,539), those with missing CRP data (n = 12,575), those without WWI data (n = 550), those with cancer (n = 903), and pregnant individuals (n = 94). After exclusions, a total of 8495 participants were included in the final analysis. Figure 1 shows the flowchart for selecting samples.

### 2.2. Variables

The exposure variable was WWI, computed by dividing WC by the square root of body weight. A mobile examination center was used to measure WC and weight, with laboratory tests carried out under controlled conditions.^[20]^ The outcome variable was CRP. CRP was measured using a 2-reagent immunoturbidimetric assay, with a lower limit of detection of 0.15 mg/L. Based on the Questionnaire Data, smoking was defined as reporting “Every day” or “Some days” and not smoking as reporting “Not at all” to the question, “Do you now smoke cigarettes?” Heavy drinking is defined as consuming ≥4 drinks per day for women and ≥5 drinks per day for men; moderate drinking is defined as consuming 2 to 3 drinks per day for women and 3 to 4 drinks per day for men; light drinking is defined as <1 drink per day for women and <2 drinks per day for men. Hypertension and diabetes were defined based on self-reported physician diagnosis.

Moreover, in this cross-sectional study, we included covariates, including age, sex, race/ethnicity, education level, marital status, ratio of family income to poverty (PIR), smoking, alcohol consumption, hypertension, diabetes mellitus, coronary heart disease, alanine aminotransferase, aspartate transaminase, alkaline phosphatase (ALP), gamma glutamyl transferase (GGT), blood urea nitrogen, serum creatinine (SCR), lactate dehydrogenase (LDH), total cholesterol, high-density lipoprotein cholesterol (HDL-C), low-density lipoprotein cholesterol, total protein, uric acid, and triglycerides. More details are available at https://www.cdc.gov/nchs/nhanes/.

### 2.3. Statistical analysis

In accordance with the analytical guidelines established by the National Center for Health Statistics, we utilized appropriate sampling weights to ensure national representation. To examine the association between WWI and CRP, 3 distinct weighted multivariable regression models were utilized. Results are presented as unstandardized beta coefficients (β) with 95% confidence intervals (CIs), indicating the mean change in CRP (mg/L) per 1-unit increase in WWI. Model 1 was unadjusted. Model 2 was adjusted for sex, age, and race/ethnicity. Model 3 was adjusted for age, sex, race/ethnicity, education level, marital status, PIR, smoking, alcohol consumption, hypertension, diabetes, coronary heart disease, alanine aminotransferase, aspartate transaminase, ALP, blood urea nitrogen, total cholesterol, HDL-C, low-density lipoprotein cholesterol, SCR, GGT, LDH, total protein, uric acid, and triglycerides. After transforming the WWI into quartile categories (quartiles), trend tests were conducted to examine the linear association between WWI and CRP. Stratified analyses were conducted to examine the association between WWI and CRP based on specific subgroup factors. To further analyze the nonlinearity in the link between WWI and CRP, weighted generalized additive models, smooth curve fittings, and threshold effects evaluation were performed. All analyses were performed using EmpowerStats software (http://www.empowerstats.com) and R software (http://www.Rproject.org), with a *P* value <.05 considered statistically significant.

### 2.4. Ethical approval and informed consent

The studies underwent rigorous review and received approval from the Institutional Research Ethics Review Board of the Centers for Disease Control and Prevention’s National Center for Health Statistics. All participants provided written informed consent. All the methods used in this study strictly followed the guiding principles of the Declaration of Helsinki. This secondary analysis did not require approval from an institutional review board.

## 3. Results

### 3.1. Baseline characteristics

Table 1 presents the weighted baseline characteristics of the 8495 individuals included in the analysis. Compared with the lowest WWI quartile, those in the highest quartile tended to be older, female, non-Hispanic White, more educated, and living with a partner. In addition, they exhibited a higher prevalence of diabetes mellitus, hypertension, and coronary heart disease. Lifestyle patterns in the highest quartile of WWI were characterized by lower smoking and alcohol consumption rates compared with the lowest quartile. In addition, a higher WWI tended to be associated with higher levels of triglycerides, uric acid, LDH, GGT, ALP, and CRP, while PIR, SCR, and HDL-C levels were lower (Table 1).

**Table 1:** Baseline characteristics of participants according to the quartiles of WWI.

WWI quartile	Q1 (lowest)	Q2	Q3	Q4 (highest)	*P* value
N	2124	2123	2124	2124	
WWI range (cm/√kg)	8.507–10.537	10.537–11.116	11.117–11.687	11.688–14.140	
Demographics					
Age (yr)	34.0 (26.0–46.0)	46.0 (34.0–58.0)	54.0 (40.0–65.0)	61.0 (48.0–70.0)	<.001
Female	837 (39.41%)	936 (44.09%)	1108 (52.17%)	1460 (68.73%)	<.001
Race/ethnicity (%)					
Mexican American	188 (8.85%)	325 (15.31%)	424 (19.96%)	468 (22.03%)	<.001
Non-Hispanic White	675 (31.78%)	631 (29.72%)	649 (30.55%)	730 (34.37%)	
Non-Hispanic Black	634 (29.85%)	436 (20.54%)	414 (19.49%)	392 (18.46%)	
Other race	627 (29.52%)	731 (34.43%)	637 (29.99%)	534 (25.14%)	
Education level (%)					
<High school	289 (13.61%)	418 (19.72%)	511 (24.07%)	669 (31.54%)	<.001
High school	469 (22.08%)	501 (23.63%)	547 (25.77%)	526 (24.80%)	
>High school	1366 (64.31%)	1201 (56.65%)	1065 (50.17%)	926 (43.66%)	
Marital status (%)					
Separated	957 (45.06%)	735 (34.64%)	743 (35.03%)	912 (42.96%)	<.001
Living with partner	1167 (54.94%)	1387 (65.36%)	1378 (64.97%)	1211 (57.04%)	
PIR	2.49 (1.38–4.18)	2.49 (1.35–3.88)	2.35 (1.19–3.67)	1.96 (1.08–2.79)	<.001
Lifestyle factors					
Current smokers (%)	481 (60.35%)	421 (47.73%)	371 (40.15%)	329 (37.30%)	<.001
Alcohol drinking (%)					
Light	763 (47.84%)	721 (49.49%)	647 (49.05%)	554 (50.41%)	.060
Moderate	561 (35.17%)	458 (31.43%)	456 (34.57%)	383 (34.85%)	
Heavy	271 (16.99%)	278 (19.08%)	216 (16.38%)	162 (14.74%)	
Laboratory parameters					
ALT (U/L)	18.00 (14.00–25.00)	21.00 (15.00–29.00)	20.00 (15.00–29.00)	20.00 (15.00–27.00)	<.001
AST (U/L)	21.00 (18.00–26.00)	22.00 (18.00–27.00)	21.00 (18.00–26.00)	21.00 (17.00–26.00)	.190
ALP (IU/L)	63.00 (52.00–77.00)	68.00 (57.00–83.00)	73.00 (60.00–89.00)	77.00 (63.00–94.00)	<.001
BUN (mmol/L)	4.64 (3.93–5.71)	5.00 (3.93–5.71)	5.00 (3.93–6.07)	5.00 (3.93–6.43)	<.001
TC (mmol/L)	4.66 (4.06–5.33)	4.99 (4.29–5.66)	4.96 (4.29–5.72)	4.84 (4.16–5.59)	<.001
HDL-C (mmol/L)	1.45 (1.19–1.78)	1.29 (1.06–1.58)	1.27 (1.06–1.55)	1.24 (1.06–1.53)	<.001
LDL-C (mmol/L)	2.84 ± 0.61	2.94 ± 0.62	2.92 ± 0.66	2.88 ± 0.63	<.001
SCR (μmol/L)	77.79 (66.30–90.17)	73.37 (61.88–86.63)	72.49 (60.11–85.75)	69.84 (58.34–84.20)	<.001
GGT (U/L)	17.00 (13.00–25.00)	21.00 (15.00–34.00)	22.00 (15.00–33.00)	22.00 (16.00–34.00)	<.001
LDH (U/L)	136.88 ± 30.70	141.94 ± 33.60	145.02 ± 32.94	149.10 ± 36.83	<.001
Total protein (g/L)	72.32 ± 4.22	72.07 ± 4.28	71.68 ± 4.54	71.35 ± 4.46	<.001
Uric acid (μmol/L)	310.14 ± 80.70	322.34 ± 86.30	326.25 ± 86.72	332.51 ± 88.90	<.001
Triglycerides (mmol/L)	0.99 (0.70–1.51)	1.36 (0.93–2.12)	1.46 (1.02–2.13)	1.59 (1.12–2.29)	<.001
CRP (mg/L)	0.90 (0.45–2.10)	1.70 (0.77–3.70)	2.30 (1.10–4.91)	3.31 (1.57–7.10)	<.001
Comorbidities, n (%)					
Hypertension	340 (16.02%)	633 (29.87%)	856 (40.36%)	1139 (53.67%)	<.001
Diabetes mellitus					
Yes	61 (2.87%)	205 (9.66%)	347 (16.35%)	600 (28.28%)	<.001
No	2029 (95.57%)	1876 (88.37%)	1699 (80.07%)	1451 (68.38%)	
Borderline	33 (1.55%)	42 (1.98%)	76 (3.58%)	71 (3.35%)	
Coronary heart disease	18 (0.85%)	37 (1.75%)	84 (3.98%)	160 (7.59%)	<.001

Values are mean ± SD/median (Q1–Q3) or n (%). *P* values were calculated using the Kruskal–Wallis test for continuous variables with non-normal distribution, the chi-square test for categorical variables, and ANOVA for continuous variables with normal distribution.

ALP = alkaline phosphatase, ALT = alanine aminotransferase, ANOVA = analysis of variance, AST = aspartate transaminase, BUN = blood urea nitrogen, CRP = C-reactive protein, GGT = gamma glutamyl transferase, HDL-C = high-density lipoprotein cholesterol, LDH = lactate dehydrogenase, LDL-C = low-density lipoprotein cholesterol, PIR = ratio of family income to poverty, SCR = serum creatinine, SD = standard deviation, TC = total cholesterol, WWI = weight-adjusted waist index.

### 3.2. Association between the WWI and CRP

The association between WWI and CRP was shown in Table 2. In the unadjusted model 1, WWI showed a significant association with CRP (β = 1.716, 95% CI = 1.549–1.883, *P* < .00001). In model 2, after adjusting for age, sex, and race/ethnicity, the association was also significant (β = 1.970, 95% CI = 1.774–2.165, *P* < .00001). The complete adjusted model 3 demonstrated a statistically significant positive correlation (β = 1.060, 95% CI = 0.652–1.468, *P* < .00001). The positive correlation persisted even after converting the WWI into quartiles (all *P* for trend < .001).

**Table 2:** Weighted linear regression analysis of the association between WWI and CRP levels (N = 8495).

Exposure	Model 1		Model 2		Model 3	
	β (95% CI)	*P* value	β (95% CI)	*P* value	β (95% CI)	*P* value
WWI (continuous)	1.716 (1.549–1.883)	<.00001	1.970 (1.774–2.165)	<.00001	1.060 (0.652–1.468)	<.00001
WWI (quantiles)						
Q1 (lowest)	Reference		Reference		Reference	
Q2	1.426 (1.049–1.803)	<.00001	1.671 (1.285–2.056)	<.00001	1.148 (0.437–1.858)	.00157
Q3	2.082 (1.695–2.469)	<.00001	2.421 (2.007–2.834)	<.00001	1.750 (0.967–2.532)	.00001
Q4 (highest)	3.960 (3.557–4.362)	<.00001	4.291 (3.837–4.745)	<.00001	2.120 (1.212–3.029)	<.00001
*P* for trend	<.001		<.001		<.001	

Model 1: unadjusted.

Model 2: adjusted for age, sex, and race/ethnicity.

Model 3: further adjusted for education level, marital status, income-to-poverty ratio, smoking, alcohol consumption, hypertension, diabetes, coronary heart disease, and laboratory parameters (ALT, AST, ALP, BUN, TC, HDL-C, LDL-C, SCR, GGT, LDH, total protein, uric acid, and triglycerides).

ALP = alkaline phosphatase, ALT = alanine aminotransferase, AST = aspartate transaminase, BUN = blood urea nitrogen, CI = confidence interval, CRP = C-reactive protein, GGT = gamma-glutamyl transferase, HDL-C = high-density lipoprotein cholesterol, LDL-C = low-density lipoprotein cholesterol, LDH = lactate dehydrogenase, SCR = serum creatinine, TC = total cholesterol, WWI = weight-adjusted waist index.

### 3.3. Subgroup analyses

Subgroup analyses were conducted stratified by sex, age, race/ethnicity, marital status, smoking, alcohol consumption, diabetes mellitus, hypertension, and coronary heart disease to evaluate the consistency of the association between WWI and CRP and to explore potential population differences (Table 3). Fully adjusted model 3 revealed a statistically significant positive association between WWI and CRP among individuals aged <60 years (β = 1.209, 95% CI = 0.749–1.669, *P* < .00001), those who were separated (β = 1.527, 95% CI = 1.021–2.033, *P* < .00001), current smokers (β = 1.311, 95% CI = 0.844–1.779, *P* < .00001), heavy drinkers (β = 1.728, 95% CI = 0.758–2.698, *P* = .00052), participants with diabetes mellitus (β = 2.282, 95% CI = 1.031–3.532, *P* = .00041), and those with coronary heart disease (β = 4.357, 95% CI = 1.461–7.253, *P* = .0043).

**Table 3:** Subgroup analysis of the association between WWI and CRP levels (N = 8495).

	Model 1		Model 2		Model 3	
	β (95% CI)	*P* value	β (95% CI)	*P* value	β (95% CI)	*P* value
Age						
<60	2.158 (1.948 to 2.367)	<.00001	2.082 (1.864 to 2.299)	<.00001	1.209 (0.749 to 1.669)	<.00001
≥60	1.028 (0.699 to 1.358)	<.00001	1.045 (0.700 to 1.391)	<.00001	0.309 (−0.495 to 1.114)	.45095
Sex						
Male	1.419 (1.193 to 1.644)	<.00001	1.695 (1.426 to 1.964)	<.00001	1.178 (0.776 to 1.580)	<.00001
Female	1.765 (1.511 to 2.020)	<.00001	2.122 (1.840 to 2.404)	<.00001	0.899 (0.100 to 1.698)	.02767
Race/ethnicity						
Mexican American	1.588 (1.030 to 2.147)	<.00001	1.336 (0.684 to 1.989)	.00006	0.861 (−1.065 to 2.787)	.38175
Other race	1.379 (1.020 to 1.739)	<.00001	1.506 (1.083 to 1.929)	<.00001	1.019 (0.176 to 1.861)	.01813
Non-Hispanic White	1.668 (1.412 to 1.925)	<.00001	1.999 (1.700 to 2.297)	<.00001	0.925 (0.318 to 1.532)	.00290
Non-Hispanic Black	2.587 (2.195 to 2.978)	<.00001	2.588 (2.116 to 3.061)	<.00001	1.650 (0.949 to 2.351)	<.00001
Marital status						
Separated	1.623 (1.362 to 1.884)	<.00001	1.922 (1.597 to 2.247)	<.00001	1.527 (1.021 to 2.033)	<.00001
Living with partner	1.797 (1.579 to 2.014)	<.00001	2.015 (1.770 to 2.261)	<.00001	0.699 (0.093 to 1.305)	.02382
Smoking						
Yes	1.782 (1.299 to 2.266)	<.00001	1.907 (1.337 to 2.476)	<.00001	1.311 (0.844 to 1.779)	<.00001
No	1.792 (1.396 to 2.188)	<.00001	1.873 (1.399 to 2.347)	<.00001	0.738 (0.071 to 1.406)	.03031
Alcohol drinking						
Light	1.341 (1.109 to 1.573)	<.00001	1.566 (1.290 to 1.843)	<.00001	0.593 (0.106 to 1.080)	.01720
Moderate	1.962 (1.630 to 2.293)	<.00001	2.039 (1.669 to 2.408)	<.00001	0.862 (0.098 to 1.626)	.02736
Heavy	2.129 (1.564 to 2.693)	<.00001	2.488 (1.842 to 3.134)	<.00001	1.728 (0.758 to 2.698)	.00052
Diabetes mellitus						
No	1.607 (1.434 to 1.780)	<.00001	1.807 (1.606 to 2.008)	<.00001	0.864 (0.427 to 1.301)	.00011
Borderline	2.879 (1.452 to 4.305)	.0001	3.012 (1.508 to 4.517)	.00012	1.412 (−2.688 to 5.511)	.50491
Yes	1.512 (0.793 to 2.231)	.00004	2.113 (1.305 to 2.920)	<.00001	2.282 (1.031 to 3.532)	.00041
Coronary heart disease						
Yes	1.402 (0.003 to 2.802)	.05051	2.438 (0.899 to 3.978)	.00209	4.357 (1.461 to 7.253)	.00430
No	1.731 (1.563 to 1.900)	<.00001	1.955 (1.758 to 2.151)	<.00001	1.016 (0.602 to 1.430)	<.00001

Model 1: unadjusted.

Model 2: adjusted for age, sex, and race/ethnicity.

Model 3: further adjusted for education level, marital status, income-to-poverty ratio, smoking, alcohol consumption, hypertension, diabetes, coronary heart disease, and laboratory parameters (ALT, AST, ALP, BUN, TC, HDL-C, LDL-C, SCR, GGT, LDH, total protein, uric acid, and triglycerides).

ALP = alkaline phosphatase, ALT = alanine aminotransferase, AST = aspartate transaminase, BUN = blood urea nitrogen, CI = confidence interval, CRP = C-reactive protein, GGT = gamma-glutamyl transferase, HDL-C = high-density lipoprotein cholesterol, LDL-C = low-density lipoprotein cholesterol, LDH = lactate dehydrogenase, SCR = serum creatinine, TC = total cholesterol, WWI = weight-adjusted waist index.

### 3.4. Threshold effect analyses

**Figure 2.**
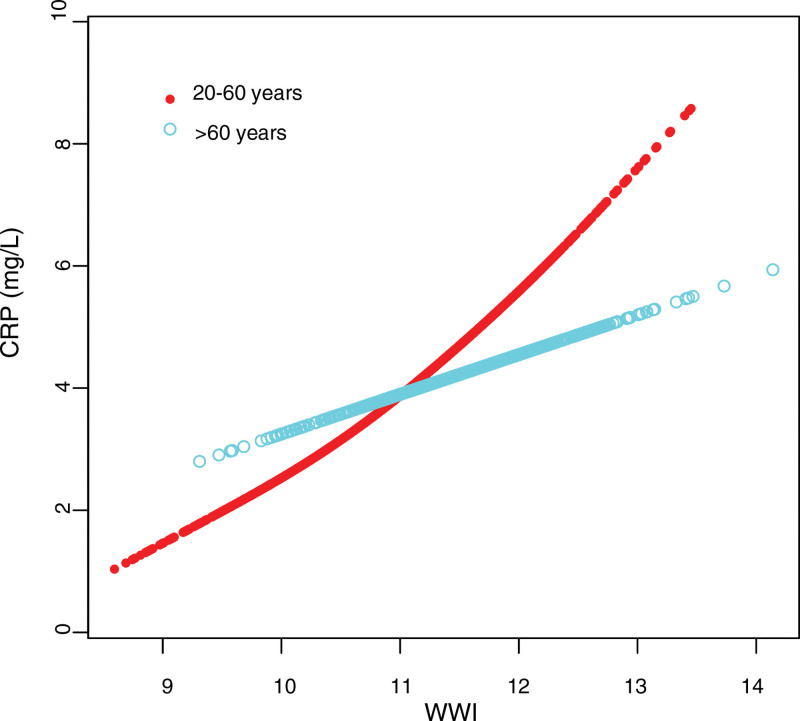
The association between WWI and CRP stratified by age. Sex, race/ethnicity, education level, marital status, PIR, smoking, alcohol consumption, hypertension, diabetes, coronary heart disease, ALT, AST, ALP, BUN, TC, HDL-C, LDL-C, SCR, GGT, LDH, total protein, uric acid, and triglycerides were adjusted. ALP = alkaline phosphatase, ALT = alanine aminotransferase, AST = aspartate transaminase, BUN = blood urea nitrogen, CRP = C-reactive protein, GGT = gamma-glutamyl transferase, HDL-C = high-density lipoprotein cholesterol, LDH = lactate dehydrogenase, LDL-C = low-density lipoprotein cholesterol, PIR = ratio of family income to poverty, SCR = serum creatinine, TC = total cholesterol, WWI = weight-adjusted waist index.

**Figure 3.**
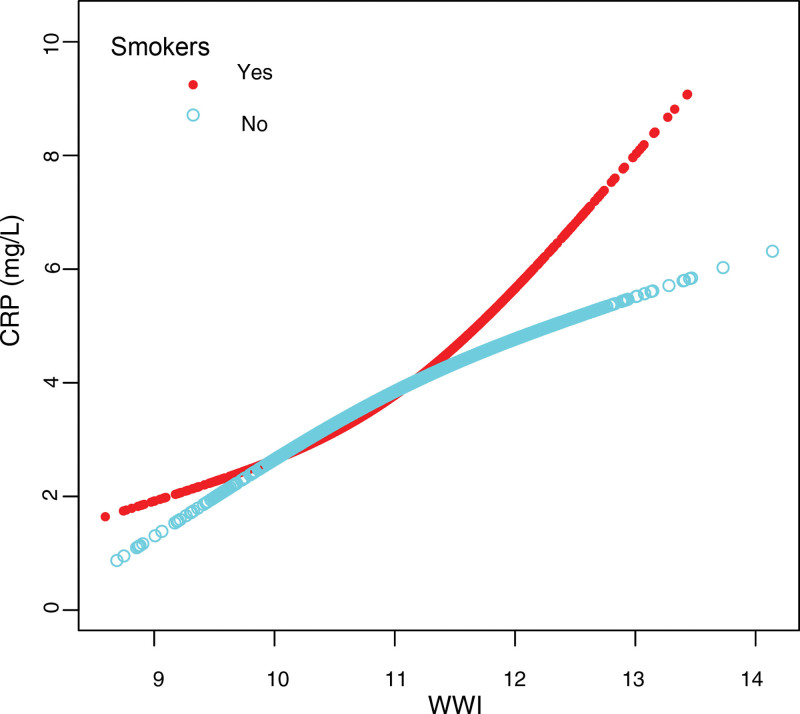
The association between WWI and CRP stratified by smoking status. Age, sex, race/ethnicity, education level, marital status, PIR, alcohol consumption, hypertension, diabetes, coronary heart disease, ALT, AST, ALP, BUN, TC, HDL-C, LDL-C, SCR, GGT, LDH, total protein, uric acid, and triglycerides were adjusted. ALP = alkaline phosphatase, ALT = alanine aminotransferase, AST = aspartate transaminase, BUN = blood urea nitrogen, CRP = C-reactive protein, GGT = gamma-glutamyl transferase, HDL-C = high-density lipoprotein cholesterol, LDH = lactate dehydrogenase, LDL-C = low-density lipoprotein cholesterol, PIR = ratio of family income to poverty, SCR = serum creatinine, TC = total cholesterol, WWI = weight-adjusted waist index.

Furthermore, generalized additive models with smooth curve fitting were employed to characterize the nonlinear association between WWI and CRP in adults aged 20 to 60 years and current smokers, as illustrated in Figures 2 and 3. Moreover, saturation effect analysis was performed in Table 4. Using a 2-piecewise linear regression model (Table 4), the inflection point for the nonlinear relationship between WWI and CRP in individuals aged 20 to 60 years was determined to be 11.514 cm/√kg. The effect sizes and CIs for the left and right sides of the inflection point were 0.790 (0.204–1.376) and 2.595 (1.326–3.864), respectively. These findings indicated that WWI significantly increased CRP levels in individuals in the 20 to 60 years group, particularly when WWI exceeded 11.514 cm/√kg. Similarly, we observed a comparable nonlinear relationship within the smoking population, with an inflection point at 12.349 cm/√kg. On the left side of the inflection point, the effect size and CI were 1.049 (0.546–1.55), whereas on the right side, they were 6.289 (2.682–9.89). These results indicated that WWI was significantly associated with CRP in the smoking population, particularly when WWI exceeded 12.349 cm/√kg.

**Table 4:** Threshold effect analysis of WWI (cm/√kg) on CRP (mg/L).

CRP	Threshold effect	
	β (95% CI)	*P* value
Subgroup analysis stratified by age		
WWI turning point for age <60 yr (K)	11.514	
<K, effect1	0.790 (0.204–1.376)	.0083
>K, effect2	2.595 (1.326–3.864)	<.0001
Log-likelihood ratio		.021
Subgroup analysis stratified by smoking		
WWI turning point for current smoker (K)	12.349	
<K, effect1	1.049 (0.546–1.552)	<.0001
>K, effect2	6.289 (2.682–9.897)	.0007
Log-likelihood ratio		.006

Age, sex, race/ethnicity, education level, marital status, PIR, smoking, alcohol consumption, hypertension, diabetes, coronary heart disease, ALT, AST, ALP, BUN, TC, HDL-C, LDL-C, SCR, GGT, LDH, total protein, uric acid, and triglycerides were adjusted.

ALP = alkaline phosphatase, ALT = alanine aminotransferase, AST = aspartate transaminase, BUN = blood urea nitrogen, CI = confidence interval, CRP = C-reactive protein, GGT = gamma-glutamyl transferase, HDL-C = high-density lipoprotein cholesterol, LDL-C = low-density lipoprotein cholesterol, LDH = lactate dehydrogenase, SCR = serum creatinine, TC = total cholesterol, WWI = weight-adjusted waist index.

## 4. Discussion

In this cross-sectional study including 8495 representative adults, we found a positive association between WWI and CRP, indicating that individuals with higher WWI were more likely to have chronic inflammation. Subgroup analyses further confirmed the robustness of this positive association across diverse population backgrounds, suggesting that increased WWI may serve as a potential marker for chronic inflammation. These findings highlight the need for further longitudinal studies to explore the role of WWI in inflammation-related health outcomes.

The World Health Organization reports that since 1990, the global prevalence of obesity among adults has more than doubled, while adolescent obesity has increased 4-fold, with over 1 billion people currently living with obesity.^[21]^ The prevalence of type 2 diabetes mellitus has also risen in parallel with the obesity epidemic, making it a significant comorbidity.^[22–24]^ Moreover, obesity is linked to increased cardiovascular mortality and morbidity.^[24,25]^ Inflammation is a key factor in the development of obesity and its related disorders, including metabolic syndrome, type 2 diabetes mellitus, cardiovascular diseases, and nonalcoholic fatty liver disease.^[26,27]^

Given the rising rates of obesity and its related disorders in contemporary society, it is essential to evaluate obesity and chronic inflammation in clinical settings. Prior research indicated a link between low-grade inflammation and higher levels of proinflammatory cytokines associated with visceral fat tissue.^[28]^ Currently, studies have revealed a positive relationship between CRP and body fat percentage.^[2]^ Another investigation indicated that CRP levels increased alongside various obesity indicators in adults.^[29]^ Although these studies did not directly examine the relationship between WWI and CRP, their findings on the inflammatory nature of adiposity indirectly support our results.

Racial disparities in inflammation, particularly the higher CRP levels observed in non-Hispanic Black participants, warrant further exploration. Our finding that non-Hispanic Black individuals had significantly higher CRP levels is consistent with previous research involving 13,517 participants, which reported that Black individuals were more likely than White individuals to exhibit elevated CRP level.^[30]^ Several factors may contribute to this disparity. From a biological perspective, the higher CRP levels observed in non-Hispanic Black participants may be partially explained by the Duffy-null genotype.^[31]^ From a social and structural standpoint, chronic stress stemming from socioeconomic disadvantage and perceived discrimination may activate neuroendocrine and inflammatory pathways, contributing to elevated CRP levels over time.^[32,33]^

Currently, the mechanisms underlying the observed positive relationship between WWI and CRP remain incompletely understood. Several potential mechanisms may contribute. First, it is well established that visceral adipose tissue is significantly infiltrated by macrophages that secrete interleukin-6. This cytokine enters the portal circulation and stimulates hepatocytes to increase the synthesis of CRP.^[34,35]^ Second, adipose tissue releases additional proinflammatory mediators, such as adiponectin, resistin, leptin, and CRP.^[36]^ Furthermore, in obesity, hypertrophic adipocyte hypertrophy and overcrowding result in cellular hypoxia and necrosis. Necrotic adipocytes recruit mononuclear macrophages, increasing their proportion in adipose tissue from 10% to 15% to 40% to 50%, which leads to macrophage infiltration.^[37]^ This infiltration stimulates macrophage polarization toward the M1 pro-inflammatory phenotype, resulting in the production and release of inflammatory mediators such as tumor necrosis factor-α, interleukin-1, and nitric oxide synthase, thereby modulating both local and systemic inflammation.^[38,39]^

Several limitations of this study should be acknowledged. First, the cross-sectional design limits our ability to establish causal relationships between WWI and CRP levels. Second, the interpretation of CRP as a marker of chronic inflammation is indirect, as elevated CRP may reflect shared risk factors with multiple conditions, including obesity, metabolic syndrome, and other inflammatory states. Third, while we adjusted for a comprehensive set of covariates, CRP levels may be influenced by factors other than WWI, including unmeasured inflammatory or immune-mediated diseases (e.g., autoimmune disorders, occult infections) for which we could not fully adjust. Fourth, the 95% CIs for several estimates were relatively wide (e.g., main effect: β = 1.060, 95% CI = 0.652–1.468), indicating limited precision. Thus, despite statistical significance, these effect sizes should be interpreted cautiously and confirmed in larger, well-powered studies. Nevertheless, this study has several significant strengths. A major strength lies in its use of National Health and Nutrition Examination Survey data obtained through a stratified multistage probability sampling approach, thereby enhancing the reliability and representativeness of our findings. Moreover, we conducted subgroup analyses to elucidate the association between WWI and CRP across different population contexts more clearly. Finally, we adjusted for confounding factors to minimize their potential impact and obtain more robust results.

## 5. Conclusion

This study suggests that WWI is positively associated with CRP, particularly among individuals with coronary heart disease, diabetes mellitus, heavy drinking, and non-Hispanic Black participants. For current smokers and individuals aged 20 to 60 years, higher WWI above the identified inflection points was associated with a steeper increase in CRP levels. However, the CIs for these estimates were relatively wide, indicating imprecision. Therefore, these findings should be interpreted cautiously, and WWI may be a tentative marker for identifying populations at potentially higher risk of inflammation, pending replication in larger studies.

## Acknowledgments

We would like to express our heartfelt thanks to all the staff members who worked on the NHANES database and for generously providing us with the necessary data free of charge.

## Author contributions

**Conceptualization:** Qiaoli Liu, Fei Wu.

**Data curation:** Caifeng Gu, Haifeng Miao.

**Formal analysis:** Caifeng Gu.

**Methodology:** Xiaofeng Zhang.

**Software:** Changcai Wu.

**Investigation:** Piao Hu.

**Supervision:** Fei Wu.

**Writing – original draft:** Qiaoli Liu.

**Writing – review & editing:** Xiaofeng Zhang.
